# LncRNA PVT1 induces apoptosis and inflammatory response of bronchial epithelial cells by regulating miR-30b-5p/BCL2L11 axis in COPD

**DOI:** 10.1186/s41021-023-00283-4

**Published:** 2023-10-10

**Authors:** Taoli Fu, Hui Tian, Hui Rong, Ping Ai, Xiaoping Li

**Affiliations:** 1 Department of Geriatrics, Wuhan Hospital of Traditional Chinese Medicine, Wuhan, 430016 Hubei China; 2 Department of Pulmonology, Wuhan Hospital of Traditional Chinese Medicine, Wuhan, 430016 Hubei China; 3 Department of Surgery, Wuhan Hospital of Traditional Chinese Medicine, Wuhan, 430016 Hubei China; 4 Department of Orthopaedics, Wuhan Hospital of Traditional Chinese Medicine, No.49, Lihuangpi Road, Jiang’an District, Wuhan, Hubei China

**Keywords:** COPD, Cigarette smoke, Inflammation, Apoptosis, PVT1

## Abstract

**Background:**

Chronic obstructive pulmonary disease (COPD) is a serious health burden worldwide with high mortality. LncRNA plasmacytoma variant translocation 1 (PVT1) has been illustrated to serve as a biomarker for COPD progression. Nonetheless, its specific functions and mechanisms in COPD are unclarified.

**Methods:**

Cigarette smoke extract (CSE) was utilized to stimulate 16HBE cells, and cigarette smoke combining with lipopolysaccharide (LPS) was employed to induce COPD in rats. Western blotting and RT-qPCR were utilized for measuring protein and RNA levels. Flow cytometry was implemented for detecting cell apoptosis. Concentrations of inflammatory factors TNF-α and IFN-γ were examined using ELISA. Luciferase reporter assay was utilized for verifying the interaction between molecules. Hematoxylin–eosin staining was performed for histological analysis of rat lung tissues.

**Results:**

PVT1 was highly expressed in CSE-stimulated 16HBE cells and the lungs of COPD rats. PVT1 depletion restored the viability, restrained apoptosis and hindered inflammatory cytokine production in 16HBE cells under CSE treatment and alleviated pathological damages in COPD rats. PVT1 bound to miR-30b-5p and miR-30b-5p targeted BCL2 like 11 (BCL2L11). Overexpressing BCL2L11 offset the above effects mediated by PVT1 in CSE-triggered 16HBE cells.

**Conclusion:**

PVT1 enhances apoptosis and inflammation of 16HBE cells under CSE stimulation by modulating miR-30b-5p/BCL2L11 axis.

**Supplementary Information:**

The online version contains supplementary material available at 10.1186/s41021-023-00283-4.

## Introduction

Chronic obstructive pulmonary disease (COPD) is a prevalent chronic disorder of the respiratory tract featured with progressive airflow obstruction and airway inflammation [1]. It has become the third leading cause of mortality worldwide [2]. COPD not only adversely affects patients’ quality of life and shortens their life expectancy, but also poses a great burden on the family and society [3]. Cigarette smoking, including passive smoking, is considered the primary inducement of COPD [4]. Inhalation of cigarette smoke (CS) containing noxious gas and particles induces inflammatory cell recruitment to lung tissues and inflammatory cytokine release, which aggravates airway inflammation and promotes cell apoptosis, contributing to the disease onset and progression [5]. Nonetheless, the molecular mechanisms underlying CS-induced airway inflammation and cell apoptosis are unclarified.

Long noncoding RNAs (lncRNAs) are a groups of transcripts with over 200 nucleotides in length that function as pivotal regulators in diverse pathophysiological processes [6]. Although with no protein-coding ability, lncRNAs can modulate gene expression via multiple mechanisms, including the competing endogenous RNA (ceRNA) network [7]. Through the ceRNA network, lncRNAs can affect mRNA stability by competitively interacting with the shared miRNAs [8]. Intriguingly, mounting evidence has suggested that dysregulation of lncRNAs is strongly related to COPD pathogenesis and development. For example, a recent report proposed that lncRNA GAS5 enhances pyroptosis by regulating miR-223-3p/NLRP3 axis in a cell model of COPD [9]. CCAT1 facilitates CS extract (CSE)-triggered inflammation in human bronchial epithelial cells by binding to miR-152-3p and promoting ERK signaling [10]. LncRNA plasmacytoma variant translocation 1 (PVT1), located on chromosome 8q24, has been extensively validated as a tumor promoter [11]. Ma et al. demonstrated that PVT1 regulates miR-149 to aggravate airway inflammation and promote cell permeability during asthma [12]. Depletion of PVT1 decreases expression of proinflammatory cytokines tumor necrosis factor (TNF)-α and interleukin (IL)-1β in macrophages under lipopolysaccharide stimulation [13]. Moreover, PVT1 is highly expressed in COPD patients and has a positive correlation with proinflammatory cytokine levels [14]. Based on the above evidence, we speculated that PVT1 is closely associated with COPD progression.

BCL2 like 11 (BCL211, also known as BIM), containing a Bcl-2 homology domain 3, exhibits a pro-apoptotic function [15]. Importantly, a previous report demonstrated that miR-9a-5p represses human pulmonary microvascular endothelial cell (HPMEC) apoptosis in COPD by downregulating BCL2L11 [16], indicating the significant role of BCL2L11 in COPD. However, whether BCL2L11 has a relation with PVT1 in COPD is unclear.

Herein, we examined lncRNA PVT1 function and its molecular mechanism in COPD. It was speculated that PVT1 promoted CSE-triggered cell apoptosis and inflammation by modulating its downstream molecules. The results might develop new therapeutic ideas for ameliorating COPD.

## Materials and methods

### Preparation of CSE

CSE was prepared based on previous report [17]. In brief, the smoke from 10 cigarettes (Hongta Tobacco Co, Ltd., Yunan, China; tar: 10 mg, nicotine: 0.8 mg, carbon monoxide: 12 mg) was bubbled through 25 mL PBS. The resulting solution was adjusted to PH7.4, filter-sterilized and designated as 100% CSE. The solution was diluted with PBS to a final concentration of 2.5% and stored at -80 °C until use. The typical chemicals in CSE include nicotine, carbon monoxide, nitric oxide, various aldehydes such as acrolein and acetaldehyde, as well as phenolic hydrocarbons such as benzopyrene, hydroquinone, resorcinol and catechol.

### Cell culture and treatment

Human bronchial epithelial cell line 16HBE from WheLab (Shanghai, China) was incubated in RPMI 1640 medium (Procell, Wuhan, China) containing 10% fetal bovine serum (FBS; Procell) and 1% penicillin/streptomycin (Procell) in a humidified atmosphere at 37 °C with 5% CO_2_. To mimic COPD, 16HBE cells were treated with 2.5% CSE for 24 h.

### Cell transfection

The short hairpin RNA targeting PVT1 (sh-PVT1) and the scrambled control (sh-NC), miR-30b-5p mimics (miR-30b-5p) and its negative control (miR-NC), BCL2L11 overexpression vector and the empty vector were obtained from GenePharma (Shanghai, China). 16HBE cells were inoculated into 6-well plates and transfected with the above plasmids using Lipofectamine 3000 (Invitrogen, Carlsbad, CA). Cells were collected for subsequent use after 24 h.

### Cell counting kit-8 (CCK-8) assay

After indicated transfection and CSE treatment, 16HBE cells were inoculated in 96-well plates (1 × 10^4^ cells/well) and treated with 10 μL CCK-8 solution (Beyotime, Shanghai, China), following by another 2-h incubation. A microplate reader (Thermo Scientific, Waltham, MA) was employed for determining the absorbance at 570 nm.

### Flow cytometry

Flow cytometry was utilized for cell apoptosis analysis using Annexin V-FITC/PI Apoptosis Detection Kit (Beyotime). 16HBE cells were washed with PBS, centrifuged at 1000 g for 5 min and resuspended in 195 μL 1 × binding buffer. Then the cells were treated with 5 μL Annexin V-FITC and 10 μL propidium iodide (PI) solution for 10 min at room temperature without light. Cell apoptosis was measured using a flow cytometer (BD Biosciences, Franklin Lakes, NJ).

### Western blotting

Protein extraction from 16HBE cells was implemented using RIPA lysis buffer (Solarbio, Beijing, China). Protein concentration was determined using a bicinchoninic acid (BCA) assay kit (Solarbio). Protein samples (20 μg) were dissolved by 10% SDS-PAGE, blotted onto polyvinylidene fluoride membranes (Beyotime) and blocked with 5% nonfat milk. The membranes were incubated at 4 °C overnight with primary antibodies against: Bax (ab182733, 1:2000), Bcl-2 (ab182858, 1:2000), cleaved (C)-caspase3 (ab2302, 1:500), BCL2L11 (ab32158, 1:500), GAPDH (ab22555, 1:1000) (all from Abcam, Shanghai, China) and then incubated with the secondary antibody (ab97080, 1:5000, Abcam) for 1 h. Blot visualization was achieved using an ECL detection kit (Solarbio).

### Luciferase reporter assay

Putative binding sites between PVT1 and miR-30b-5p or miR-30b-5p and BCL2L11 were shown by the ENCORI or TargetScan databases, respectively. The predicted wide-type binding sequences on PVT1 or BCL2L11 3’UTR and the mutated sequences were separately inserted into pmirGLO vector (Promega, Madison, WI). These constructs were co-transfected with miR-30b-5p or miR-NC into 16HBE cells. After 48 h, a Dual Luciferase Reporter Assay System (Beyotime) was employed for luciferase activity assessment.

### Animal models

Forty male Wistar rats (12 weeks, 200 ± 20 g) were obtained from Hubei Experimental Animal Center (Wuhan, China) and housed in SPF environments. This study was approved by the Ethics Committee of Wuhan Hospital of Traditional Chinese Medicine and all animal experiments were implemented following the NIH Guide for the Care and Use of Laboratory Animals.

Lentivirus expressing sh-PVT1 (LV-sh-PVT1) and its control LV-NC were synthesized by GenePharma. The rats were randomly grouped as follows: 1) sham group, 2) COPD group, 3) COPD + LV-NC group, 4) COPD + LV-sh-PVT1 group, with 10 rats per group. As previously described [18], a COPD rat model was constructed by combining CS with lipopolysaccharide (LPS). Briefly, after anesthesia, all rats, except those in the sham group, received intratracheal instillation of LPS (200 μg/kg, Sigma-Aldrich, St. Louis, MO) on days 1, 15 and 28 and were exposed to CS for 30 min in a homemade smoking box from day 2 to day 27 (except day 15). Rats in the sham group received normal saline and the room air with the same procedure. After modeling, LV-sh-PVT1 or LV-NC (2 × 10^7^ TU in 50 μL) were injected into COPD rats via tail vein for knocking down PVT1.

### Specimen collection

Fifteen days after lentivirus injection, blood samples were collected from rat orbits and centrifuged at 3500 rpm for 10 min to obtain serum. Then, all rats were sacrificed under anesthesia, and the broncho-alveolar lavage fluid (BALF) was collected by inserting a cannula into the tracheas of rats. The lungs were gently rinsed three times with sterilized normal saline. The collected BALF was centrifuged at 4 °C at 3000 rpm for 10 min. The supernatants were collected, and protein concentration was measured using BCA method (Solarbio). The lung tissues were collected for histological examination or measurement of gene expression.

### Hematoxylin–eosin (HE) staining

Fresh lung tissues were fixed in 4% paraformaldehyde, paraffin-embedded and sliced (4-μm-thick). Next, tissue slices were dewaxed in xylene for 5 min, rehydrated in gradient ethanol and soaked in ddH_2_O for 2 min. Afterwards, the sections were stained with hematoxylin (Solarbio) for 15 min, treated with 5% acetic acid and rinsed twice with tap water, followed by staining with eosin for 2 min. After dehydration in graded ethanol, transparentizing by xylene and seal with neutral resin (Sigma-Aldrich), the lung tissues were observed under a microscope (Olympus, Tokyo, Japan).

### Real time quantitative polymerase chain reaction (RT-qPCR)

Total RNA isolation from 16HBE cells or rat lung tissues was performed using TRIzol reagent (Invitrogen). cDNA was prepared using iScript cDNA Synthesis Kit (Bio-Rad, Hercules, CA). RT-qPCR was implemented using SYBR Premix Ex Taq II kit (Takara, Dalian, China) on a SimpliAmp™ PCR System (Thermo Scientific). With U6 or GAPDH as normalization, relative gene expression was measured using the 2^−ΔΔCt^ method. Primer sequences were shown in Table S1.

### Enzyme-linked immunosorbent assay (ELISA)

Concentrations of IFN-γ and TNF-α in 16HBE cell culture supernatant and rat serum were respectively determined using human IFN-γ ELISA kit (ab46025), human TNF-α ELISA kit (ab181421), rat IFN-γ ELISA kit (ab46107) and rat TNF-α ELISA kit (ab46070) (all from Abcam) following the manufacturer’s protocols.

### Statistical analysis

Data are expressed as mean ± standard deviation. Each experiment was repeated in triplicate. Student’s *t*-test or one-way ANOVA were used for difference comparisons among groups using SPSS 25.0 software (IBM, Armonk, NY), and *p*<0.05 indicated statistical significance.

## Results

### PVT1 silencing attenuates CSE-triggered 16HBE cell apoptosis and inflammation

**Fig. 1:**
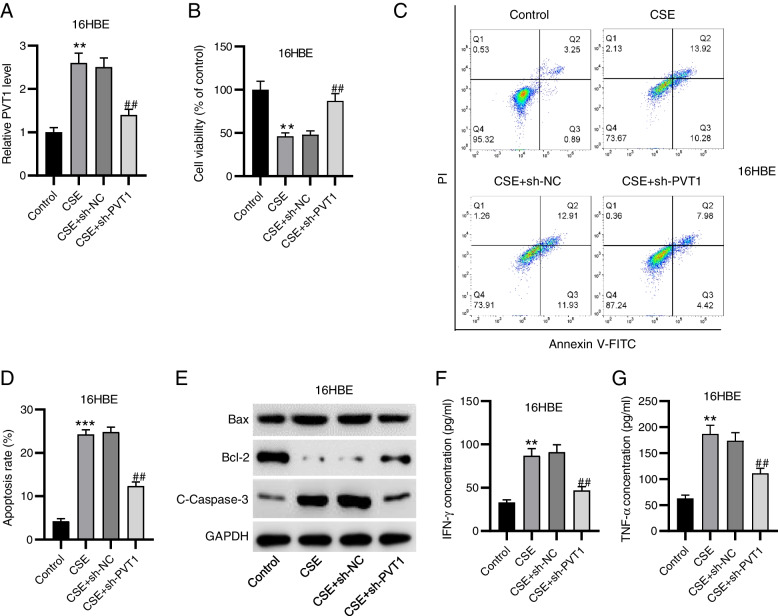
PVT1 silencing attenuates CSE-triggered 16HBE cell apoptosis and inflammation. **A** RT-qPCR analysis of PVT1 expression in 16HBE cells with indicated treatments. **B** CCK-8 assay for evaluating cell viability. **C**, **D** Flow cytometry for detecting cell apoptosis. **E** Western blotting for measuring levels of apoptosis-related proteins. **F**, **G** ELISA for determining concentrations of IFN-γ and TNF-α. ^**^p<0.01, ^***^p<0.001 vs. control group; ^##^*p*<0.01 vs. CSE + sh-NC group

The 16HBE cells were exposed to CSE and transfected with sh-PVT1. Notably, CSE-treated cells displayed a markedly higher level of PVT1, and sh-PVT1 transfection decreased its high level in CSE-stimulated cells (Fig. 1A), indicating that PVT1 was successfully silenced. We then examined PVT1 effect on cell viability, apoptosis and inflammation. As depicted by CCK-8 assay, PVT1 silencing prominently abated CSE-evoked reduction in cell viability (Fig. 1B). Moreover, flow cytometry demonstrated that CSE stimulation significantly facilitated 16HBE cell apoptosis, whereas the effect was offset by knocking down PVT1 (Fig. 1C, D). To confirm this, we measured apoptosis-associated protein levels in 16HBE cells with different treatments. Consistently, the results depicted that PVT1 downregulation markedly counteracted CSE-triggered increase in Bax and C-caspase3 protein levels and decrease in Bcl-2 level (Fig. 1E). Furthermore, we evaluated PVT1 influence on CSE-induced inflammation by detecting concentrations of inflammatory factors IFN-γ and TNF-α. Notably, CSE treatment increased their levels in 16HBE cells, while PVT1 depletion abated these effects (Fig. 1F, G), as demonstrated by ELISA. Collectively, PVT1 silencing alleviates CSE-triggered 16 HBE cell apoptosis and inflammation.

### PVT1 binds to miR-30b-5p

**Fig. 2:**
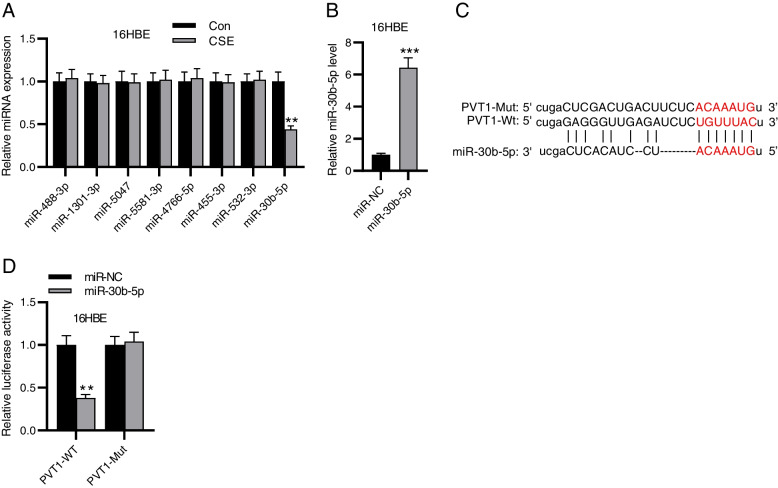
PVT1 binds to miR-30b-5p. **A** RT-qPCR for evaluating expression of predicted miRNAs in 16HBE cells with or without CSE treatment. **B** RT-qPCR for elucidating miR-30b-5p overexpression efficiency in 16HBE cells. **C** ENCORI predicts the binding sequence of miR-30b-5p on PVT1. **D** Luciferase reporter assay for verifying the interaction between miR-30b-5p and PVT1. ^**^p<0.01, ^***^p<0.001

Subsequently, we searched PVT1 downstream miRNAs using the ENCORI database. With the screening conditions of CLIP Data ≥ 2 and Degradome Data ≥ 1, eight candidate miRNAs were predicted (Table S2). Among these eight miRNAs, only miR-30b-5p was prominently downregulated in 16HBE cells under CSE treatment (Fig. 2A). Thus, miR-30b-5p was selected for further analysis. We overexpressed miR-30b-5p in 16HBE cells (Fig. 2B). To elucidate the interaction between miR-30b-5p and PVT1, we mutated the binding sequence on PVT1 predicted from the ENCORI database (Fig. 2C). Relative to miR-NC, miR-30b-5p overexpression prominently weakened the luciferase activity of PVT1 but had no marked impact on that of the mutated type (Fig. 2D), validating that PVT1 could bind to miR-30b-5p in 16HBE cells.

### miR-30b-5p targets BCL2L11

**Fig. 3:**
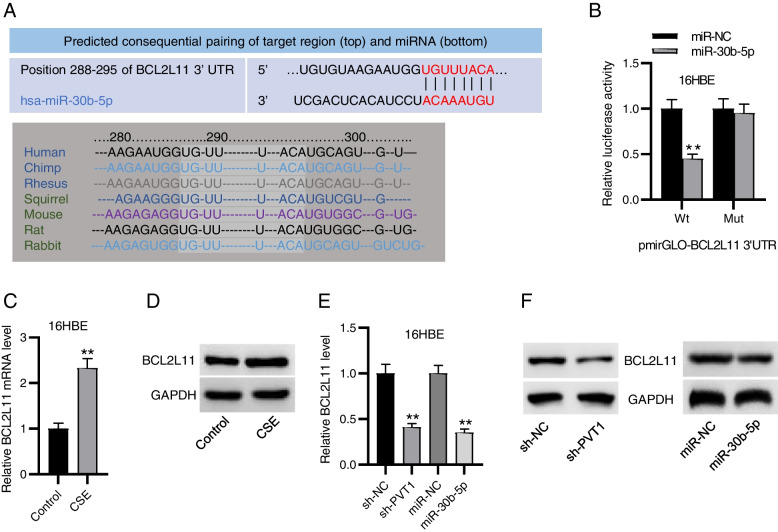
miR-30b-5p targets BCL2L11. **A** TargetScan database predicts the binding site between miR-30b-5p and BCL2L11. **B** Luciferase reporter assay for elucidating the interaction between miR-30b-5p and BCL2L11. **C**, **D** RT-qPCR and western blotting for detecting BCL2L11 mRNA (**C**) and protein expression (**D**) in 16HBE cells with or without CSE exposure. **E**, **F**. RT-qPCR and western blotting for analyzing BCL2L11 mRNA (**E**) and protein expression (**F**) in 16HBE cells with PVT1 knockdown or miR-30b-5p overexpression. ^**^p<0.01

Numerous reports have suggested that lncRNAs interact with miRNAs to indirectly affect downstream mRNA stability [8]. TargetScan predicts the binding site (red marked) between miR-30b-5p and BCL2L11 3’UTR, and the predicted binding sequence on BCL2L11 (light grey highlighting) was highly conserved across various species (Fig. 3A). Notably, the luciferase activity of BCL2L11 was prominently reduced in miR-30b-5p-overexpressed 16HBE cells but was almost unaltered after mutation (Fig. 3B), suggesting that miR-30b-5p directly targeted BCL2L11. We also tested BCL2L11 expression in 16HBE cells under CSE treatment. The results depicted that BCL2L11 mRNA expression was much higher in CSE-exposed cells than that in the controls (Fig. 3C). This was further confirmed by western blotting (Fig. 3D). Moreover, knocking down PVT1 or overexpressing miR-30b-5p significantly repressed BCL2L11 mRNA and protein expression (Fig. 3E, F). The results indicated that PVT1 could upregulate BCL2L11 expression by competitively interacting with miR-30b-5p.

### Overexpressing BCL2L11 abates sh-PVT1-mediated effects on 16HBE cell apoptosis and inflammation under CSE treatment

**Fig. 4:**
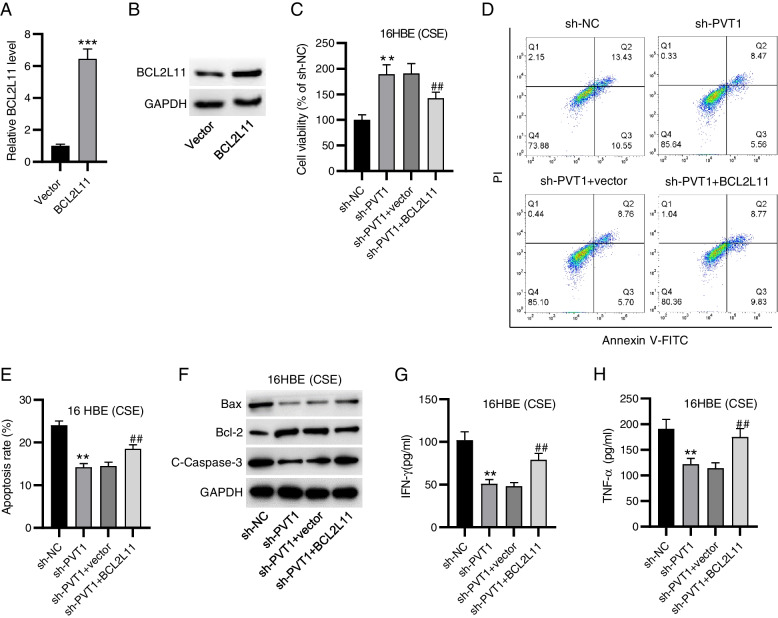
Overexpressing BCL2L11 abates sh-PVT1-mediated effects on 16HBE cell apoptosis and inflammation under CSE treatment. **A**-**B** RT-qPCR and western blotting for determining BCL2L11 overexpression efficiency. **C** CCK-8 for evaluating the viability of 16HBE cells with indicated treatments. **D**, **E** Flow cytometry for measuring cell apoptosis. **F** Western blotting for detecting apoptosis-related proteins. **G**, **H** ELISA for examining concentrations of IFN-γ and TNF-α. ^**^p<0.01, ^***^p<0.001 vs. sh-NC group; ^##^*p*<0.01 vs. sh-PVT1 + vector group

Rescue experiments were implemented to validate whether PVT1 regulated CSE-triggered 16HBE cell inflammation and apoptosis by modulating miR-30b-5p/BCL2L11 axis. BCL2L11 overexpression vector was transfected into 16HBE cells, followed by evaluation of BCL2L11 expression. Notably, BCL2L11 mRNA level was markedly elevated in BCL2L11-overexpressed cells (Fig. 4A). Western blotting displayed the consistent results (Fig. 4B), confirming that BCL2L11 was successfully overexpressed. As expected, overexpressing BCL2L11 reversed PVT1 silencing-triggered improvement in the viability of CSE-exposed 16HBE cells (Fig. 4C). In parallel, PVT1 knockdown-mediated decrease in cell apoptosis was offset by BCL2L11 overexpression under CSE stimulation, as demonstrated by flow cytometry (Fig. 4D, E). Consistently, upregulating BCL2L11 counteracted sh-PVT1-evoked elevation in Bcl-2 protein expression and reduction in Bax and C-caspase3 protein levels in 16HBE cells under CSE stimulation (Fig. 4F). Likewise, PVT1 depletion-triggered decrease in production of cytokines IFN-γ and TNF-α was partially reversed by BCL2L11 upregulation (Fig. 4G, H). Collectively, the above results suggested that PVT1 promoted CSE-triggered 16HBE cell inflammation and apoptosis by upregulating BCL2L11.

### PVT1 depletion ameliorates COPD in rats

**Fig. 5:**
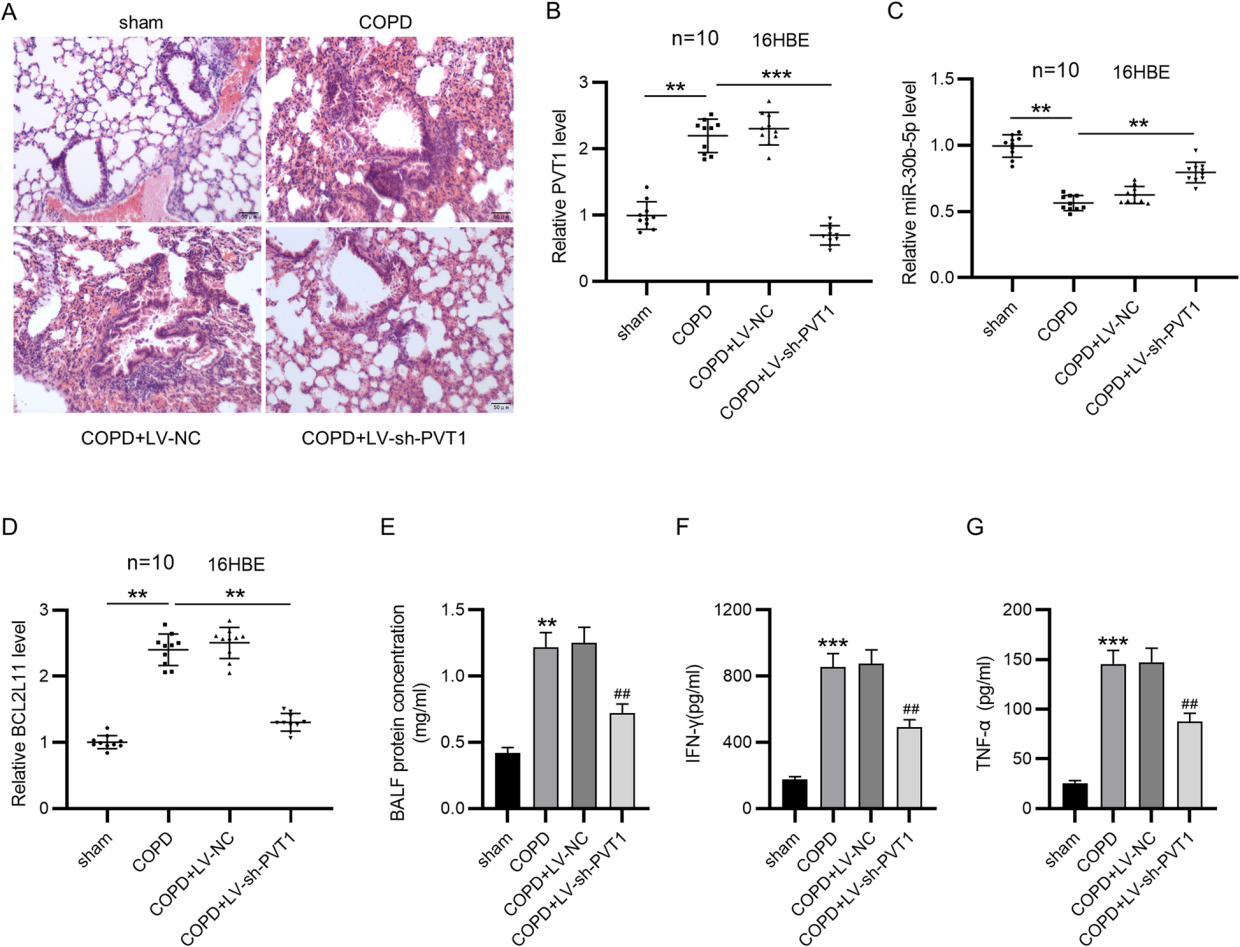
PVT1 depletion ameliorates COPD in rats. **A** Representative images of HE staining for histological observation of rat lung tissues of each group. **B**-**D** RT-qPCR for determining expression of PVT1 (**B**), miR-30b-5p (**C**) and BCL2L11 (**D**) in rat lung tissues. **E** Measurement of BALF protein concentration. **F**, **G** ELISA for examining concentrations of IFN-γ and TNF-α in the serum of rats. ^**^p<0.01, ^***^p<0.001; ^##^*p*<0.01 vs. COPD + LV-NC group

To further elucidate PVT1 function in COPD, we established a rat COPD model via CS plus LPS exposure, and lentivirus carrying sh-PVT1 (LV-sh-PVT1) was injected into COPD rats. As depicted by HE staining, relative to the control group, the COPD as well as COPD + LV-NC groups exhibited inflammatory cell infiltration, thinning of alveolar all, lumen deformation and alveolar cavity enlargement. However, knocking down PVT1 alleviated the above conditions in the lung tissues of COPD rats (Fig. 5A). We then detected PVT1, miR-30b-5p and BCL2L11 expression in the lungs of each group. Consistent with the in vitro results described above, PVT1 expression was prominently higher in COPD rats than the sham-operated rats, whereas injection of LV-sh-PVT1 decreased its high expression in COPD rats (Fig. 5B). Conversely, relative to the sham group, the COPD and COPD + LV-NC groups displayed a markedly decreased level of miR-30b-5p, which was counteracted by PVT1 depletion (Fig. 5C). Similar to that of PVT1, the expression of BCL2L11 was upregulated in COPD rats and was decreased by PVT1 knockdown (Fig. 5D). These indicated that PVT1 sponged miR-30b-5p to upregulated BCL2L11 in the lungs of COPD rats. Moreover, as displayed in Fig. 5E, knocking down PVT1 markedly decreased the high protein concentration in BALF of COPD rats. ELISA revealed that serum levels of IFN-γ and TNF-α were elevated in COPD rats, while PVT1 silencing significantly abated these effects (Fig. 5F, G). Taken together, PVT1 regulated BCL2L11 expression via miR-30b-5p and depletion of PVT1 could ameliorate COPD in rats.

## Discussion

COPD is a serious health burden globally. The existing treatments for COPD can effectively improve lung functions and prevent acute exacerbation, however, they bring tremendous economic burden and have little impact on mortality [19, 20]. CS exposure is the most critical risk factor for COPD. Harmful substances in CS induces airway remodeling that involves inflammation, apoptosis, oxidative stress and other pathological alterations, ultimately leading to progressive and irreversible airflow obstruction [21]. HBE cells, a first site of contact for environmental stimuli, is critical for maintaining normal airway function [22]. Based on the previous evidence, we established a CSE-triggered 16HBE cell model and a rat COPD model via combination of CS exposure and LPS for exploring lncRNA PVT1 functions in COPD.

Previous reports have illustrated that PVT1 exhibits a pivotal role in multiple diseases, such as osteoarthritis, epilepsy and peripheral nerve injury [23–25]. Importantly, PVT1 displays a high level in COPD patients and has a positive correlation with the serum levels of inflammatory cytokines [14]. Consistently, our results depicted that PVT1 was highly expressed in CSE-stimulated 16HBE cells and in the lungs of COPD rat models relative to corresponding controls. Functionally, knocking down PVT1 prominently restored the viability, restrained apoptosis and decreased concentrations of inflammatory factors in 16HBE cells under CSE treatment. Previous evidence has illustrated that PVT1 exerts a promoting effect on cell apoptosis and inflammation in epilepsy [24]. PVT1 knockdown facilitates viability and repress apoptosis of high-glucose-treated ARPE-19 cells [26]. These reports support our findings described above. Furthermore, animal experiments displayed that depletion of PVT1 alleviated the pathological changes COPD rat lungs and decreased the high serum levels of TNF-α and IFN-γ. Elevation of TNF-α and IFN-γ serum concentrations has been observed in COPD patients [27]. Collectively, the above results indicated that PVT1 might promote COPD progression.

It is well documented that lncRNAs can work as miRNA sponges to indirectly affect mRNA stability and translation [8]. PVT1 has also been shown to work as a molecular sponge of miRNAs in multiple disorders [24, 26]. Here, to explore potential mechanism underlying the pro-COPD effect of PVT1, we searched its downstream miRNAs via bioinformatics analysis. As validated by the assays, PVT1 could bind to miR-30b-5p in 16BHE cells. Our results showed that miR-30b-5p was downregulated in the lungs of COPD rats, indicating its potential role in COPD progression. Previous studies have shown that PVT1 can interact with many miRNAs, such as miR-152, miR-128-3p and miR-194-5p [28–30]. Intriguingly, a previous study has demonstrated that PVT1 is negatively correlated with miR-146a in COPD patients and miR-146a predicts reduced COPD susceptibility and decreased acute exacerbation risk [14]. However, these known interactions, including PVT1-miR-146a, were not detected in the present study using the ENCORI database, which may be attributed to the limitation of the selected screening conditions as well as the limitation of the database itself. Future studies need to utilize additional databases to elucidate the interactions of PVT1 with its downstream miRNAs. Moreover, further investigations are needed to clarify whether miR-146a or miR-30b-5p plays a more important role in the pro-COPD effect of PVT1. As miRNAs exert their biological functions through modulating their downstream targets, future studies may benefit from data concerning the downstream targets of miR-146a in COPD.

Furthermore, our study revealed that miR-30b-5p targeted BCL2L11, a BCL-2 family member, and that BCL2L11 was upregulated in the lungs of COPD rats. Additionally, we confirmed that PVT1 upregulated BCL2L11 expression by sponging miR-30b-5p in COPD rat models. Rescue experiments revealed that overexpressing BCL2L11 counteracted PVT1 knockdown-mediated restoration of the viability, repression of apoptosis and decrease of inflammatory cytokine production in CSE-induced 16HBE cells, indicating that PVT1 regulated COPD progression in vitro via the miR-30b-5p/BCL2L11 axis. A previous report demonstrated that lncRNA TUG1 promotes CSE-induced apoptosis of HPMECs by regulating the miR-9a-5p/BCL2L11 axis [16], confirming the pro-apoptotic effect of BLC2L11.

In conclusion, this study displays that knocking down lncRNA PVT1 represses apoptosis and inflammation in 16HBE cells under CSE stimulation and ameliorates COPD in rat models probably by modulating miR-30b-5p/BCL2L11 axis. Our research may help to develop a new strategy for COPD treatment.

### Supplementary Information

**Additional file 1:** **Table S1. **Primers used for RT-qPCR. **Table S2. **Predicted downstream miRNAs of PVT1 from ENCORI database. 
