# Correction: LncRNA PVT1 induces apoptosis and inflammatory response of bronchial epithelial cells by regulating miR-30b-5p/BCL2L11 axis in COPD

**DOI:** 10.1186/s41021-024-00309-5

**Published:** 2024-07-18

**Authors:** Taoli Fu, Hui Tian, Hui Rong, Ping Ai, Xiaoping Li

**Affiliations:** 1 Department of Geriatrics, Wuhan Hospital of Traditional Chinese Medicine, Wuhan, 430016 Hubei China; 2 Department of Pulmonology, Wuhan Hospital of Traditional Chinese Medicine, Wuhan, 430016 Hubei China; 3 Department of Surgery, Wuhan Hospital of Traditional Chinese Medicine, Wuhan, 430016 Hubei China; 4 Department of Orthopaedics, Wuhan Hospital of Traditional Chinese Medicine, No.49, Lihuangpi Road, Jiang’an District, Wuhan, Hubei China

**Correction: Genes and Environment (2023) 45:24**

10.1186/s41021-023-00283-4

Following publication of the original article [1], the authors found that there were some mistakes in the data for images of HE staining, and then replicated experiments and histological observations were performed again. The correct Fig. 5 has been provided in this Correction. The conclusion of the study remains intact after the replacement of these images.

The incorrect Fig. 5 is:

**Fig. 5:**
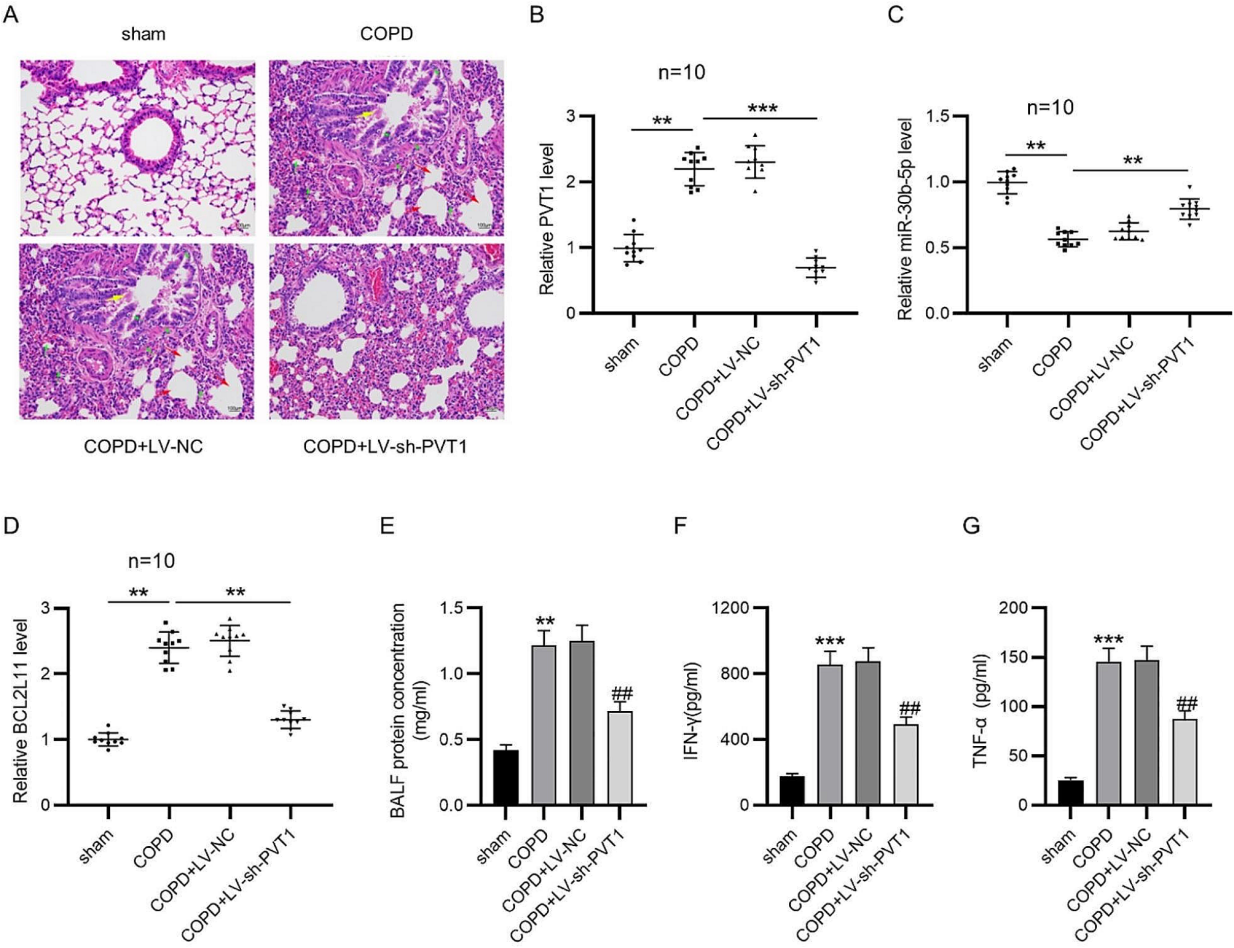
PVT1 depletion ameliorates COPD in rats. **A** Representative images of HE staining for histological observation of rat lung tissues of each group. B-D RT-qPCR for determining expression of PVT1 (**B**), miR-30b-5p (**C**) and BCL2L11 (**D**) in rat lung tissues. **E** Measurement of BALF protein concentration. **F, G** ELISA for examining concentrations of IFN-γ and TNF-α in the serum of rats. **p < 0.01, ***p < 0.001; ^##^*p* < 0.01 vs. COPD + LV-NC group

The correct Fig. 5 is:

**Fig. 5:**
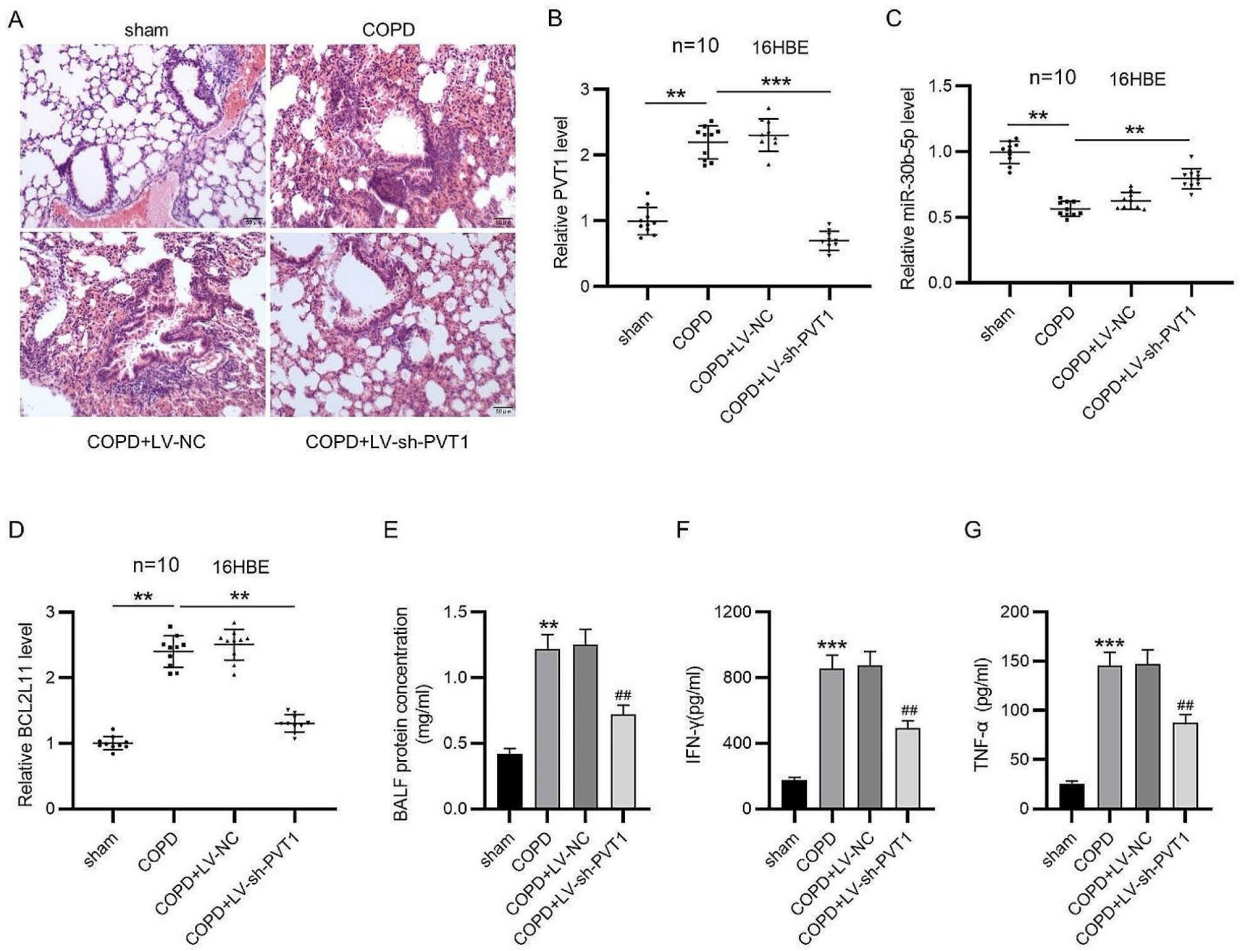
PVT1 depletion ameliorates COPD in rats. **A** Representative images of HE staining for histological observation of rat lung tissues of each group. B-D RT-qPCR for determining expression of PVT1 (**B**), miR-30b-5p (**C**) and BCL2L11 (**D**) in rat lung tissues. **E** Measurement of BALF protein concentration. **F, G** ELISA for examining concentrations of IFN-γ and TNF-α in the serum of rats. **p < 0.01, ***p < 0.001; ^##^*p* < 0.01 vs. COPD + LV-NC group

The original article [1] has been corrected.
