# Supplementary material for: LncRNA PVT1 induces apoptosis and inflammatory response of bronchial epithelial cells by regulating miR-30b-5p/BCL2L11 axis in COPD
Source: Genes Environ. 2023 Oct 10;45:24. doi: 10.1186/s41021-023-00283-4 (PMC10566077; doi:10.1186/s41021-023-00283-4)
Supplement: Supplementary file 1 — Additional file 1: Table S1. Primers used for RT-qPCR. Table S2. Predicted downstream miRNAs of PVT1 from ENCORI database. [file 41021_2023_283_MOESM1_ESM.docx]

**Table S1. Primers used for RT-qPCR.**

| Gene | Sequence (5’-3’) | |
| --- | --- | --- |
| PVT1 | Forward | CTTGCGGAAAGGATGTTGGC |
|  | Reverse | GCCATCTTGAGGGGCATCTT |
| BCL2L11 | Forward | AGACAGAGCCACAAGACAG |
|  | Reverse | ATAGTGGTTGAAGGCCTGG |
| miR-30b-5p | Forward | CCGAAACATCCTACACTCAGCTAA |
|  | Reverse | CAGTGCGTGTCGTGGAGT |
| miR-488-3p | Forward | GCGCTTGAAAGGCTGTTTC |
|  | Reverse | GTGCAGGGTCCGAGGT |
| miR-1301-3p | Forward | GCCGAGTTGCAGCTGCCTGGGA |
|  | Reverse | CTCAACTGGTGTCGTGGA |
| miR-5047 | Forward | GCCTAGACGAGACACAGTGC |
|  | Reverse | GCCAAGACCTTACAACCGCA |
| miR-5581-3p | Forward | CGTCTTGCAGGCCGTCATG |
|  | Reverse | GCTGTCAACGATACGCTACCTA |
| miR-4766-5p | Forward | CAGACATACTTTATCATCCCTT |
|  | Reverse | ACAATGCCACCTCCTCC |
| miR-455-3p | Forward | GCAGTCCATGGGCATATACAC |
|  | Reverse | GCAGGGTCCGAGGTATTC |
| miR-532-3p | Forward | GGCTTGCAGTCGTATCCAGT |
|  | Reverse | GTATCCAGTGCGTGTCGTGG |
| U6 | Forward | ATACAGAGAAAGTTAGCACGG |
|  | Reverse | GGAATGCTTCAAAGAGTTGTG |
| GAPDH | Forward | TCAAGATCATCAGCAATGCC |
|  | Reverse | CGATACCAAAGTTGTCATGGA |

**Table S2. Predicted downstream miRNAs of PVT1 from ENCORI database.**

| miRNAid | miRNAname | geneName | clipExpNum | degraExpNum |
| --- | --- | --- | --- | --- |
| MIMAT0004763 | hsa-miR-488-3p | PVT1 | 2 | 4 |
| MIMAT0005797 | hsa-miR-1301-3p | PVT1 | 4 | 1 |
| MIMAT0020541 | hsa-miR-5047 | PVT1 | 2 | 1 |
| MIMAT0022276 | hsa-miR-5581-3p | PVT1 | 2 | 1 |
| MIMAT0019917 | hsa-miR-4766-5p | PVT1 | 2 | 1 |
| MIMAT0004784 | hsa-miR-455-3p | PVT1 | 2 | 1 |
| MIMAT0004780 | hsa-miR-532-3p | PVT1 | 2 | 1 |
| MIMAT0000420 | hsa-miR-30b-5p | PVT1 | 2 | 1 |
